# Mast Cells Exert Anti-Inflammatory Effects in an IL10^−/−^ Model of Spontaneous Colitis

**DOI:** 10.1155/2018/7817360

**Published:** 2018-04-17

**Authors:** E. M. Lennon, L. B. Borst, L. L. Edwards, A. J. Moeser

**Affiliations:** ^1^Department of Small Animal Clinical Sciences, University of Tennessee College of Veterinary Medicine, Knoxville, TN 37996, USA; ^2^Department of Population Health and Pathobiology, North Carolina State University College of Veterinary Medicine, Raleigh, NC 27607, USA; ^3^Gastrointestinal Stress Biology Laboratory, Department of Large Animal Clinical Sciences, College of Veterinary Medicine, Michigan State University, East Lansing, MI 48824, USA

## Abstract

Mast cells are well established as divergent modulators of inflammation and immunosuppression, but their role in inflammatory bowel disease (IBD) remains to be fully defined. While previous studies have demonstrated a proinflammatory role for mast cells in acute models of chemical colitis, more recent investigations have shown that mast cell deficiency can exacerbate inflammation in spontaneous colitis models, thus suggesting a potential anti-inflammatory role of mast cells in IBD. Here, we tested the hypothesis that in chronic, spontaneous colitis, mast cells are protective. We compared colitis and intestinal barrier function in IL10^−/−^ mice to mast cell deficient/IL10^−/−^ (double knockout (DKO): Kit^Wsh/Wsh^ × IL10^−/−^) mice. Compared with IL10^−/−^ mice, DKO mice exhibited more severe colitis as assessed by increased colitis scores, mucosal hypertrophy, intestinal permeability, and colonic cytokine production. PCR array analyses demonstrated enhanced expression of numerous cytokine and chemokine genes and downregulation of anti-inflammatory genes (e.g., *Tgfb2*, *Bmp2*, *Bmp4*, *Bmp6*, and *Bmp7*) in the colonic mucosa of DKO mice. Systemic reconstitution of DKO mice with bone marrow-derived mast cells resulted in significant amelioration of IL10^−/−^-mediated colitis and intestinal barrier injury. Together, the results presented here demonstrate that mast cells exert anti-inflammatory properties in an established model of chronic, spontaneous IBD. Given the previously established proinflammatory role of mast cells in acute chemical colitis models, the present findings provide new insight into the divergent roles of mast cells in modulating inflammation during different stages of colitis. Further investigation of the mechanism of the anti-inflammatory role of the mast cells may elucidate novel therapies.

## 1. Introduction

IBD is an incurable, debilitating intestinal disease, and treatment often consists of lifelong systemic immunosuppression [1–3]. The pathogenesis of IBD is complex and is thought to result from a combined effect of genetic predisposition, intestinal epithelial barrier dysfunction, alterations in the intestinal microbiota, and immune dysregulation [4–7]. The adaptive immune system has been the focus of IBD research for years; more recently, research from genome-wide association studies and other immunological research has highlighted the critical role of the innate immune system [8–11]. Mast cells are innate immune cells, which are well-known for their proinflammatory roles in conditions such as allergy/anaphylaxis and asthma [12–14]. Several lines of evidence have implicated mast cells in the pathophysiology of IBD as they are found to be increased in number and exhibit a degranulated appearance in patients with Crohn's disease and ulcerative colitis [15–24]. Additionally, IBD patients demonstrate increased mast cell mediator release upon stimulation compared to controls [25–28] and have increased levels of a histamine metabolite in their urine [29]. In line with the role of mast cells in inflammatory conditions such as allergy and asthma, mast cells have been proposed to be major drivers of intestinal inflammation through various mechanisms [30–34]. However, studies investigating the role of mast cells in animal models of colitis have been conflicting. In chemical colitis models such as dextran sodium sulfate- (DSS-) and trinitrobenzene sulfonic acid- (TNBS-) induced colitis, genetic mast cell deficiency or pharmacologic inhibition of mast cells ameliorated colitis [30, 35–41], while in another, mast cell deficiency had no effect on colitis [42]. In addition, mast cell proteases increase in serum and tissue in response to DSS colitis in mice [35], and it was shown that mast cell protease-6-deficient mice exhibit dampened DSS and TNBS colitis [30]. Pharmacological mast cell stabilization (ketotifen) and tryptase and chymase inhibition (nafamostat and NK3201, resp.) decrease mucosal damage in TNBS colitis [37–40].

Together, there is strong evidence supporting a deleterious (proinflammatory) role for mast cells in acute or subacute chemical colitis. However, there are a number of studies indicating that mast cells may also play beneficial roles. For example, in a DSS colitis model in rats, an increase in mast cell numbers and mast cell protease II was observed during the resolution phases of intestinal inflammatory and histological lesions, suggesting that mast cells might play a beneficial role in the chronic or recovery phase of colitis [43]. In another study in DSS colitis, mast cell-deficient mice had delayed recovery compared to their wild-type counterparts [44]. In addition, compared with IL10-deficient mice, IL10-deficient/mast cell-deficient double-knockout mice (DKO mice) exhibit increased colitis and associated colonic pathophysiology indicating that in spontaneous colitis models, mast cells may be protective [45, 46].

While well-known for their proinflammatory properties, it has become increasingly evident that mast cells can have potent anti-inflammatory or immunosuppressive effects either via direct or indirect mechanisms in certain disease conditions [47, 48]. Mast cells have been shown to promote tolerance through induction of regulatory T cells and influence dendritic cells [49–53]. Mast cell tryptase, a serine protease, has been shown to degrade cytokines and matrix metalloproteinases and aids in bacterial defense [54]. Additionally, mast cells are important for tissue healing [55].

Mast cells mediate their functional role in homeostasis and in disease through the release of granule-associated preformed mediators (e.g., histamine, proteases, and TNF) as well as de novo-synthesized cytokines, chemokine, and lipid-derived mediators [48, 56–58]. Mast cell mediators can have diverse effects on numerous cell types and tissues including permeability of epithelial and endothelial barriers, immune cell recruitment and pathogen defense, nerve activation and sensitization, secretion, motility, and blood flow [33, 59–70].

Although deleterious in chemical colitis, mast cells may have a different effect in other colitis models that more closely replicate human IBD, for example, the IL10^−/−^ mouse. A severe form of childhood-onset IBD results from polymorphisms in the IL10 gene or that of the Il10 receptor [71–74], which is a similar pathogenesis to the IL10^−/−^ mouse. While it has been shown that mast cell deficiency enhanced spontaneous colitis in the IL10^−/−^ murine model [45, 46], the precise role for mast cells in spontaneous colitis has yet to be fully determined. Utilizing mast cell-deficient × IL10-deficient (DKO) murine model of spontaneous colitis, combined with systemic mast cell engraftment approaches, the objective of this study was to define the role of the mast cell in spontaneous colitis.

## 2. Materials and Methods

### 2.1. Experimental Groups

IL10^−/−^ mice on a C57Bl/6 background (IL10^−/−^) and IL10^−/−^ × Kit^Wsh/Wsh^ double-knockout (DKO) mice that lack mast cells in addition to IL10 (C57Bl/6 background) were used in this study. Mice were housed in a barrier facility under specific pathogen-free conditions. Among others, murine parvovirus, norovirus, and *Helicobacter* were excluded from this facility. Groups of DKO mice were reconstituted with bone marrow-derived mast cells at 4 weeks of age as previously described. Tissue collection was performed at 20–24 weeks of age [75]. Roughly equal (within *n* = 2) numbers of male and female mice were used in each experiment. Mice were killed by CO_2_ inhalation at 20–24 weeks of age.

### 2.2. Differentiation and Reconstitution of Bone Marrow-Derived Mast Cells

**Figure 1:**
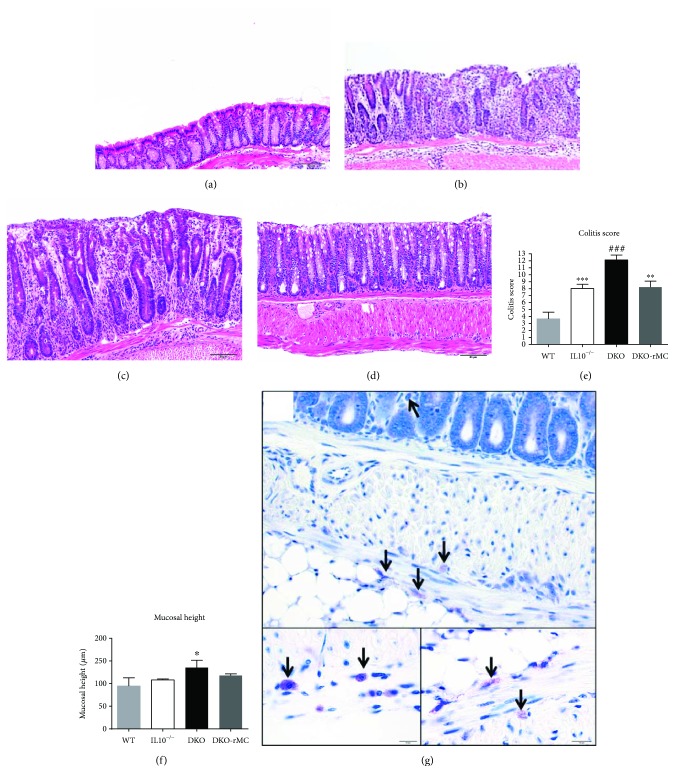
Inflammation is exacerbated in the absence of mast cells and prevented by reconstitution with bone marrow-derived mast cells. Representative H&E-stained paraffin sections of the colon of 14-to-16-week-old wild-type C57Bl/6 (a), IL10^−/−^ (b), mast cell-deficient colitis prone double knockout (DKO) (c), and DKO mice reconstituted with wild-type mast cells (DKO-rMC) (d). Colitis scores (e) and colonic mucosal height (f) are higher in DKO mice compared to IL10^−/−^, and mast cell reconstitution restores the colitis to the level of IL10^−/−^ mice. Toluidine blue-stained sections confirming the reconstitution of tissue MCs (arrows) (g). WT: *n* = 5; IL10^−/−^: *n* = 25; DKO: *n* = 25; DKO-rMC: *n* = 13. ^∗∗∗^, ^###^ *p* < 0.001; ^∗^ *p* < 0.05, ^∗∗^ *p* < 0.01 versus DKO.

Bone marrow-derived mast cells (BMMCs) were generated as previously described [75]. Briefly, cells were collected immediately postmortem by flushing bone marrow from the femur of mice. These cells were cultured in the presence of IL3 (5 ng/ml) and stem cell factor (5 ng/ml) (R&D Systems) for 8 weeks with weekly culture media changes. Mast cell purity was assessed by toluidine blue staining and confirmed by performing staining for c-kit and Fc*ε*R1 with analysis by flow cytometry, which is typically >98% in our laboratory using these procedures. DKO mice were reconstituted with BMMCs at 4 weeks of age by intraperitoneal injection of 1 × 10^7^ cells as previously described [75]. Mast cell reconstitution was performed with BMMCs derived from both wild-type and IL10^−/−^ mice to demonstrate whether the anti-inflammatory activity of the mast cell is dependent on IL10. Successful reconstitution was confirmed histologically at the time of tissue collection (16–20 weeks following reconstitution) by microscopic examination of toluidine blue-stained sections of the intestine (Figure 1). Reconstituted mice that did not have identifiable mast cells within any section of the colon were excluded from analysis.

### 2.3. Colitis Scoring

Colonic tissue sections were fixed in 10% buffered formalin and embedded in paraffin, and 4 *μ*m-thick sections were stained with hematoxylin and eosin using standard techniques. Spontaneous colitis scoring was adapted from the criteria reported by Berg et al., as previously described [76]. Five sections of hematoxylin/eosin-stained intestine were examined using a clinical light microscope (Olympus BX45), equipped with a high-resolution digital camera (Olympus DP2-BSW, version 2.2), and scored on a scale from 0–4 by a single-blinded veterinary pathologist (LBB). Mucosal (epithelial) hypertrophy was assessed by measuring the colonic epithelial thickness in micrometers. Well-oriented crypts from 5 separate colon sections per mouse and 6 randomly selected mucosal areas within each section were measured from the basement membrane of the crypt base to the mucosal section using the line tool and the 20x objective. The histological scoring criteria are provided in Supplementary Materials.

### 2.4. Assessment of Intestinal Barrier Function

#### 2.4.1. Ussing Chambers

Segments of the jejunum and proximal colon were harvested and immediately placed in oxygenated (95% O_2_ to 5% CO_2_) Ringer solution. Tissues were then mounted in 0.3 cm^2^ aperture Ussing chambers (Physiologic Instruments Inc., San Diego, CA) as described previously [77]. Mucosal-to-serosal flux of FITC-dextran (4 kDa; Sigma-Aldrich, St. Louis, MO) (FD4) was assessed as an index of paracellular permeability. After a 15-minute equilibration, FD4 (0.25 mM) was added to the mucosal side of Ussing chamber-mounted tissues. The FD4 was allowed to equilibrate for 15 minutes, after which 100 ml samples (in triplicate) were collected from the serosal side of tissues at 15-minute intervals and transferred into a 96-well assay plate. The presence of FD4 was assayed by measurement of fluorescence intensity using an fMax fluorescence microplate reader (Molecular Devices, Sunnyvale, CA), and concentrations were determined from standard curves generated by serial dilution of FD4.

#### 2.4.2. *In Vivo* FD4 Permeability

*In vivo* FD4 intestinal permeability was assessed as previously described [78]. Briefly, food was removed from mice 4 hours prior to the beginning of the study. Mice were gavaged with 30 mg/mouse FD4. Four hours after administration, serum was collected, and fluorescence intensity was assessed as described above.

### 2.5. Colonic Explant Culture and Cytokine ELISA

Colonic sections were collected and processed as previously described [79]. Colonic tissue samples were weighed, then cut into small fragments and incubated for 24 hours in cell culture media at 37°C, 5% CO_2_. Supernatants were collected and stored at −80°C until analysis. IL12p40, IL6, and TNF concentrations were determined in colonic supernatant samples using commercially available sandwich ELISA kits (BD Biosciences, Franklin Lake, NJ), and results were corrected for the amount of tissue in each well.

### 2.6. Real-Time PCR Array for Mouse Cytokines/Chemokines

RNA was extracted from rinsed colon samples that had been snap frozen in liquid nitrogen and stored at −80°C. Tissues were homogenized, and RNA was extracted using a commercially available kit (RNeasy, Qiagen, Valencia, CA) and was analyzed with a spectrophotometer. RNA was subjected to DNase treatment (RNase-free DNAse kit, Qiagen, Valencia, CA) and then was reverse transcribed using a commercially available kit (RT2 First Strand, Qiagen, Valencia, CA) followed by PCR amplification. Samples were analyzed using the RT2 Profiler Array for Mouse Cytokines/Chemokines (Qiagen, Cat number PAMM-150Z, Valencia, CA) according to the manufacturer's instructions in a LightCycler 480 (Roche Life Sciences, Indianapolis, IN) to quantify expression of genes encoding 82 mouse inflammatory cytokines and chemokines. Gene expression was normalized to five housekeeping genes included with each experiment. PCR controls and RT controls were included with each experiment. Data were analyzed, and fold changes were calculated using commercially available software (SA Biosciences, http://pcrdataanalysis.sabiosciences.com/pcr/arrayanalysis.php website).

### 2.7. Statistical Analysis

Statistical analysis was accomplished using GraphPad Prism. Groups were compared using a one-way ANOVA, and Bonferroni correction was used to control for multiple comparisons. PCR array data was analyzed using the SA Biosciences PCR array analysis software.

### 2.8. Ethical Considerations

All animals were housed in accordance with guidelines from the American Association for Laboratory Animal Care and Research Protocols, and experiments were approved by the Institutional Animal Care and Use Committee of North Carolina State University where all animal experiments were performed.

## 3. Results

### 3.1. Mast Cells Are Protective against Spontaneous Colitis

To define the role of the mast cell in spontaneous colitis, we examined colonic histopathology in 4 groups of mice on a C57/Bl6 background: wild-type (WT) mice, IL10^−/−^ mice, DKO mice, and DKO mice that were reconstituted with BMMCs. Compared with WT mice and consistent with previous reports, including our own previous study, of IL10^−/−^ mice on the C57Bl/6 background, IL10^−/−^ mice displayed mild, patchy colitis with incomplete penetrance (Figures 1(a), 1(b), and 1(e)) [76, 80, 81]. Compared with IL10^−/−^ mice, DKO mice exhibited more severe colitis by histology (Figures 1(c) and 1(e)), greater colitis scores (colitis scores = 8.0 ± 0.6 versus 12.1 ± 0.7 in IL10^−/−^ and DKO mice, respectively; *p* < 0.001, Figure 1(e)), and mucosal hypertrophy (Figure 1(f)). The colitis in DKO mice was characterized histologically by diffuse inflammatory cell infiltrates (Figure 1(c)), compared to smaller aggregates of lymphoid cells in IL10^−/−^ mice. The inflammation consisted primarily of infiltration of lymphoid cells, with few neutrophils. In some mice, the inflammatory cell infiltrate crossed through the muscularis layer of the intestine (Figure 1(c)). In order to demonstrate that the exacerbation in colitis in DKO mice was mast cell specific, DKO mice were engrafted with BMMCs from WT mice, and colitis was assessed. DKO engrafted with BMMCs reduced histopathology, colitis scores, and mucosal hypertrophy to levels of IL10^−/−^ mice (Figures 1(d)–1(f)).

**Figure 2:**
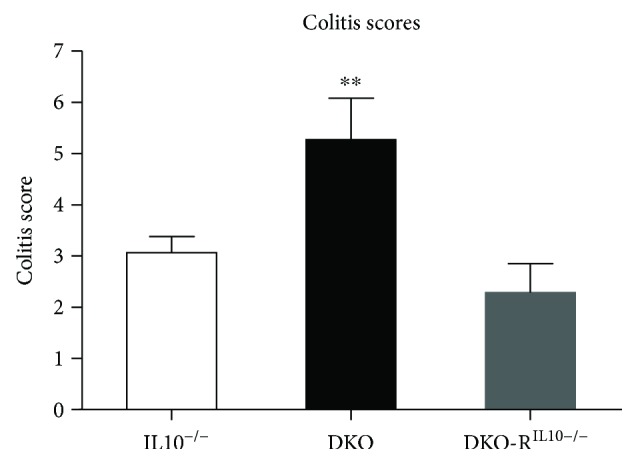
Mast cell-mediated protection against colitis is not IL10-dependent. Reconstitution of DKO mice with IL10^−/−^ bone marrow-derived mast cells (DKO-R^IL10−/−^) prevents colitis, similar to reconstitution with wild-type mast cells. IL10^−/−^: *n* = 11; DKO: *n* = 11; DKO-R^IL10−/−^: *n* = 8. ^∗∗^ *p* < 0.01.

To confirm that the protective effects of mast cell engraftment were not a result of supplying IL10 derived from engrafted WT BMMCs, we also assessed colitis in DKO mice engrafted with IL10^−/−^ BMMCs (derived from IL10^−/−^ mice). These experiments showed that engraftment of DKO mice with IL10^−/−^ BMMCs ameliorated colitis in a similar manner to WT BMMCs (IL10^−/−^ mean colitis score 3.1 ± 0.3; DKO colitis score 5.3 ± 0.8; and DKO-R^IL10−/−^ colitis score 2.2 ± 0.6; *p* < 0.005), overall indicating that mast cells' protective effects are via a mechanism independent of IL10 secretion (Figure 2).

### 3.2. Intestinal Permeability Is Elevated in DKO Mice and Is Reversed by Systemic Reconstitution of Bone Marrow-Derived Mast Cells

**Figure 3:**
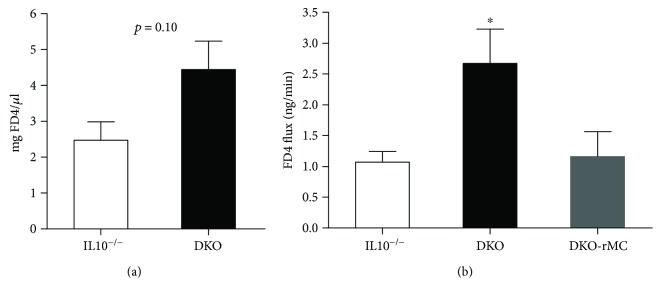
DKO mice have increased intestinal permeability that is ameliorated by reconstitution of bone marrow-derived mast cells. (a) In vivo intestinal permeability in IL10^−/−^ and DKO mice (*n* = 3–5 per group). (b) FD4 permeability across distal colonic mucosa mounted on Ussing chambers. IL10^−/−^: *n* = 23; DKO: *n* = 24; DKO-R^IL10−/−^: *n* = 10. ^∗^ *p* < 0.05.

Given that intestinal permeability is a central mechanism driving intestinal inflammation in colitis models, we next assessed the impact of mast cell deficiency on intestinal barrier function in IL10^−/−^ and DKO mice. Using a FD4 oral gavage method to assess in vivo intestinal permeability, we showed a trend (*p* = 0.10) for elevated intestinal permeability in DKO mice (Figure 3(a)) compared with IL10^−/−^ mice. Further, we assessed intestinal permeability on isolated small intestinal and distal colon segments on Ussing chambers. Consistent with the worsened colitis, DKO mice had significantly increased intestinal permeability (2.7 ± 0.6 ng/min) compared to IL10^−/−^ mice (1.1 ± 0.2 ng/min) (Figure 3(b)). Compared with DKO mice, DKO mice engrafted with BMMCs exhibited a significant reduction in intestinal permeability to levels similar to IL10^−/−^ mice.

### 3.3. Influence of Mast Cell Deficiency on Proinflammatory Cytokine Production in IL10^−/−^ Model of Colitis

**Figure 4:**
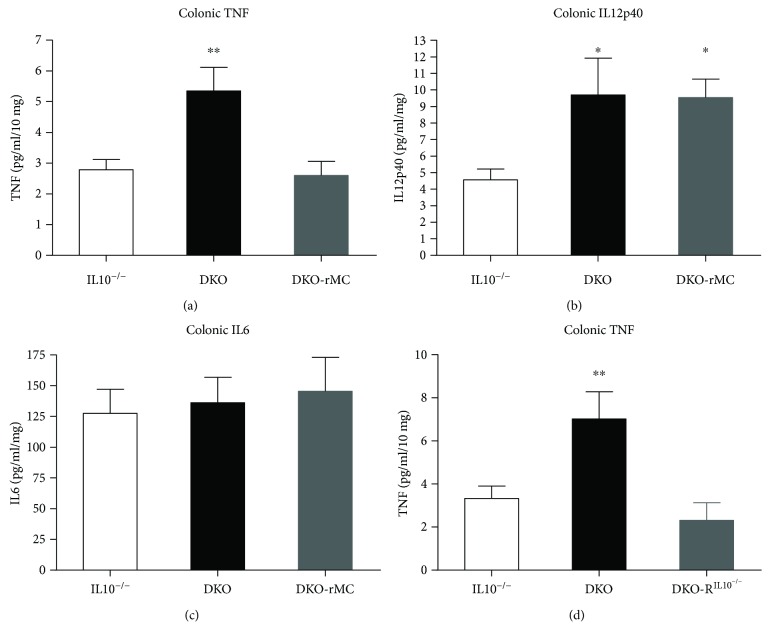
Spontaneous colonic cytokine production. (a) TNF is increased in DKO mice compared to IL10^−/−^ and is restored to baseline in DKO mice that are reconstituted with BMMCs. (b) IL12p40 is increased in DKO mice compared to IL10^−/−^ but is unaffected by BMMC reconstitution. (c) Colonic IL6 is similar between IL10^−/−^, DKO, and DKO mice reconstituted with BMMCs. (d) TNF is also restored to baseline in DKO mice reconstituted with IL10^−/−^ BMMCs compared to DKO mice. IL10^−/−^: *n* = 25; DKO: *n* = 25; DKO-rMC: *n* = 13. ^∗^ *p* < 0.05, ^∗∗^ *p* < 0.01.

The spontaneous production of proinflammatory cytokine was measured via ELISA in colonic explant supernatants. Compared with IL10^−/−^ mice, DKO had significantly elevated spontaneous colonic production of IL12p40 (4.6 ± 0.6 pg/ml/mg versus 9.7 ± 2.2 pg/ml/mg; *p* < 0.05) and TNF (2.8 ± 0.3 pg/ml/mg versus 5.3 ± 0.7 pg/ml/mg; *p* < 0.005) (Figures 4(a) and 4(b)). Engraftment of DKO mice with wild-type BMMCs ameliorated the increased TNF but not IL12p40. IL6 levels were similar between IL10^−/−^, DKO, and engrafted DKO mice (Figure 4(c)). Engraftment of DKO mice with IL10^−/−^ BMMCs ameliorated increased TNF, similar to those engrafted with wild-type BMMCs (Figure 4(d)).

### 3.4. Mast Cell Deficiency and Reconstitution in IL10^−/−^-Associated Colitis Drive the Gene Expression of an Array of Colonic Mucosal Cytokine and Chemokines

**Figure 5:**
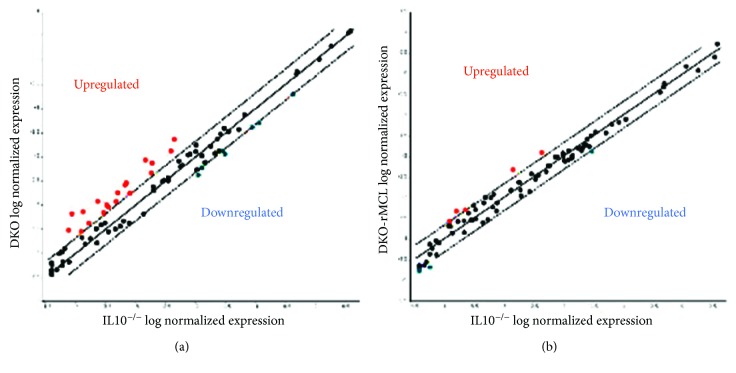
Mast cell deficiency in colitis results in dysregulation of many cytokines and chemokines. (a) Scatter plot representation of average fold change of 84 different cytokine and chemokine genes in IL10^−/−^ compared to DKO mice from RNA extracted from whole colon. Dots above the lines are higher in DKO than in IL10^−/−^ mice, and dots below the line are greater than 2-fold decreased in DKO compared to IL10^−/−^ mice. (b) Reconstitution of DKO mice with BMMCs ameliorates the alteration of cytokines. Represents averaged fold change values in *n* = 5 mice per group.

**Figure 6:**
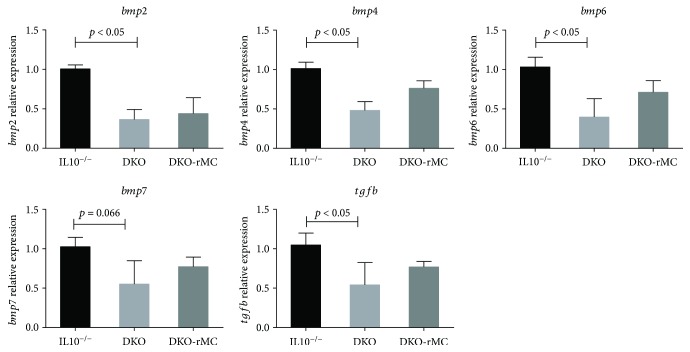
Relative expression of *bmp* genes and *tgfb* genes in colonic mucosa as influenced by mast cell deficiency and systemic reconstitution. Genes are expressed relative to IL10^−/−^ mice values as described in the PCR array material and methods.

To assess the effect of mast cell deficiency on global cytokine regulation, a real-time PCR array assessing 84 cytokines and chemokines was performed on colon mucosal samples from IL10^−/−^ and DKO mice. Comparisons between IL10^−/−^ and DKO mice demonstrated that lack of mast cells in DKO mice produced a large number of perturbations in cytokines and chemokines (Figure 5(a)) which were largely restored to IL10^−/−^ levels in DKO mice engrafted with mast cells (Figure 5(b)). Results of the cytokine alterations are displayed in Table 1. Specifically, gene expression of anti-inflammatory mediators including *tgfb* and bone morphogenetic proteins (BMPs) 2, 4, and 7 was significantly decreased in DKO mice compared with IL10^−/−^ mice and was rescued by systemic reconstitution of DKO mice with bone marrow-derived mast cells (Table 1 and Figure 6), thus demonstrating a direct role of mast cells in modulating *bmp* genes. Genes that were most highly upregulated in DKO mice were *CCl5* (by 5.19-fold), *Cd40lg* (by 2.54-fold), and *Cxcl9* (by 5.89-fold).

## 4. Discussion

The results from this study highlight a novel protective role for mast cells in a spontaneous, chronic model of colitis, in the IL10^−/−^ mouse. While the present study confirms previous reports of heightened colitis in DKO mice [45, 46], the amelioration of the colitis with systemic mast cell engraftment in DKO mice in the present study provided a definitive anti-inflammatory role for mast cells in spontaneous, chronic colitis.

Here, we demonstrated that engraftment of DKO mice with either WT or IL10^−/−^ BMMCs resulted in comparable anti-inflammatory effects, confirming that mast cell-specific IL10 was not the major anti-inflammatory mechanism. TNF is an established mediator of inflammation in the IL10^−/−^ mouse model [82] and in human IBD, in which anti-TNF monoclonal antibodies are a clinical mainstay [82, 83]. In the present study, DKO mice had elevated colonic production of TNF and histologic colitis scores that were restored to IL10^−/−^ levels with systemic reconstitution of DKO mice with BMMCs. However, mast cell reconstitution did not reduce the elevated IL6 or IL12p40, indicating that mast cells may be specifically regulating specific cytokines and (or) proinflammatory mediators to dampen colitis. The mechanism by which mast cells dampen colitis is unknown, but that ability of mast cells to suppress immune responses is well established. There are several lines of evidence that mast cells perform important immunoregulatory functions. Mast cells control T cell polarization by affecting cytokine production from other antigen-presenting cells. For example, mast cell mediators including histamine and PGD2 suppress production of IL12 from dendritic cells in vitro [84, 85]. This effect can alter T cell polarization and redirect immune responses. Mast cells also have direct effects on T cells, such as induction of T regulatory cells, which suppress inflammation [51–53, 86]. In a model of graft-versus-host disease, mast cells are essential for maintaining tolerance and preventing graft rejection via induction of T regulatory cells as well as via an unknown mechanism independent of T regulatory cells [51, 87]. Additionally, mast cell-induced T regulatory cells help to suppress inflammation in a model of allergic airway disease [88]. Mast cell mediators contained in their granules also have anti-inflammatory effects. For example, mast cell proteases, such as tryptase and chymase, degrade proinflammatory cytokines and alarmins, which results in an immunoregulatory effect [89–92]. Heparin, another mast cell mediator, has powerful anti-inflammatory effects. Heparin binds leukocyte and platelet selectins and downregulates intercellular adhesion molecule-1 (ICAM-1), preventing leukocyte migration to target tissues [93–95], and also downregulates proinflammatory cytokine expression in a process that requires its sulfate groups [96]. Mast cells can also have immunoregulatory roles by limiting pathogenic infections. Mast cells are critical for induction of protective immune responses against viral, bacterial, and parasitic pathogens. Mast cells are critical in the recruitment of neutrophils and enhance resistance to bacterial pathogens including *Klebsiella pneumonia* and *Mycoplasma pneumonia* [62, 97–99], and in the absence of mast cells, pathogen clearance in the urinary bladder is impaired [100]. Lastly, mast cells are important for tissue healing; mast cell-deficient mice have delayed wound healing compared to controls [55, 101]. Mast cells play important functions in each of the three stages of wound healing, including recruitment of neutrophils to the wound during the inflammatory phase [101, 102], stimulation of fibroblasts during the proliferative phase [103], and promoting tissue granulation, cell migration, and angiogenesis during the remodeling phase [104, 105]. Mast cell-derived proteases have been shown to degrade TNF and inflammation [51, 89].

We had hypothesized that the cytokine/chemokine array would reveal skewing toward a so-called Th1 or Th17 cytokine profile, with decreases in the Th2-type cytokines that are classically thought of as being present in mast cell activation, such as IL4 and IL5. Surprisingly, these cytokines were not significantly altered in mast cell deficiency, and a strongly Th1 or Th17 profile was not evident. Instead, we were able to identify particular mediators that were most impacted by mast cell deficiency in the setting of colitis.

Intestinal barrier dysfunction is an important mechanism in the pathogenesis of inflammation in IBD. In the present study, DKO mice exhibited increased intestinal permeability compared to IL10^−/−^ mice which has been reported previously [45]. Given that mast cell mediators such as proteases and TNF are well-known to directly increase intestinal permeability [33, 59, 60, 106, 107], the increased permeability in DKO mice is likely secondary to the increased severity of DKO colitis. Mast cell reconstitution suppressed colitis and reduced intestinal permeability, further supporting the indirect effect of mast cells on intestinal permeability in this model.

A prominent histopathological finding associated with exacerbated colitis in DKO mice was the pronounced increase in colonic mucosal height. The etiology of mucosal hypertrophy in conjunction with colitis is unknown but is a common finding that increases with colitis severity. Mucosal heights in DKO mice were restored to IL10^−/−^ levels upon mast cell reconstitution, demonstrating that mast cells were important in the resolution of mucosal hypertrophy.

While numerous cytokines and chemokines were dysregulated in DKO mice and restored by mast cell reconstitution as assessed by the cytokine array, some of the most significantly altered genes were the BMP proteins. BMP-2, -4, and -6 were all significantly decreased in DKO mice compared to IL10^−/−^. BMP proteins have an established role as modulators of inflammation in many organs. BMP-2, -4, and -7 have been demonstrated to have anti-inflammatory activity in the stomach of mice, and BMP-6 and -7 are protective in renal fibrosis. In line with the present study, BMP7 was shown to exert an anti-inflammatory effect in chemical colitis models [108, 109]. The anti-inflammatory mechanism of the BMPs is not completely understood but has been demonstrated to decrease proliferation in the Jurkat T cell line and to polarize monocytes towards M2 macrophages [110–112]. Given that the mast cell-deficient DKO mice exhibited suppressed BMP genes, which were restored with mast cell reconstitution, it is demonstrated that mast cells have a major influence on BMP gene transcription which could be a potential mechanism by which mast cells exert anti-inflammatory mechanisms. More definitive studies are required to establish this link.

## 5. Conclusions

In summary, we have demonstrated that mast cells have a key protective role in chronic spontaneous colitis. This finding is in contrast to models of chemical colitis, demonstrating that mast cells can impart protective properties during colitis in the IL10^−/−^ model. One explanation for the divergent effects of mast cells between chemical colitis and the IL10^−/−^ model is that chemical colitis models are acute (days) or subacute (weeks) disease models, while the IL10^−/−^ model more closely recapitulates the chronic, progressive, spontaneous onset of naturally occurring IBD with chronic changes to the intestinal microbiota. Therefore, consistent with reports in other body systems, it seems that while mast cells have deleterious effects in the acute or subacute setting, they have beneficial roles in chronic inflammatory diseases, potentially due to their effects that promote healing [113]. Therefore, given that naturally occurring IBD is a spontaneous, chronic disease with a slow onset, the current work represents a paradigm shift that may more accurately recapitulate the role of the mast cell in naturally occurring disease and thus direct therapeutic interventions towards mast cell activation versus mast cell blockade.

## Figures and Tables

**Table 1:** Mast cell deficiency results in dysregulation of many different cytokines and chemokines. Fold change and *p* value of DKO and DKO-rMC mice compared to IL10^−/−^. Significantly upregulated genes and downregulated genes indicated by bold and italic font, respectively (*p* = ≤0.1).

	Fold change and *p* value compared to IL10			
	DKO		DKO-rMC	
	Fold change	*p* value	Fold change	*p* value
*Adipoq*	0.93	0.605923	0.44	0.12537
*Bmp2*	*0.36*	*0.00275*	*0.43*	*0.00352*
*Bmp4*	*0.47*	*0.003545*	0.78	0.08664
*Bmp6*	*0.38*	*0.027679*	0.71	0.12534
*Bmp7*	*0.55*	*0.0662*	0.77	0.13341
*Ccl1*	**2.3**	**0.06215**	1.71	0.20753
*Ccl11*	0.54	0.157722	0.79	0.31294
*Ccl12*	*0.46*	*0.020304*	1.11	0.57195
*Ccl17*	1.12	0.862792	1.05	0.85839
*Ccl19*	1.4	0.281436	1.24	0.4384
*Ccl2*	1.46	0.268872	1.33	0.25702
*Ccl20*	0.84	0.90886	1.09	0.73897
*Ccl22*	1.23	0.36045	0.79	0.52381
*Ccl24*	0.86	0.287519	*0.55*	*0.02211*
*Ccl3*	2.5	0.304203	1.62	0.10923
*Ccl4*	3.6	0.132224	**2.05**	**0.00244**
*Ccl5*	**5.19**	**0.059787**	**2.87**	**0.02972**
*Ccl7*	1.27	0.259336	1.22	0.24594
*Cd40lg*	**2.54**	**0.035975**	**2.89**	**0.01671**
*Cd70*	0.94	0.73583	0.74	0.21501
*Cntf*	1.05	0.869816	0.79	0.11255
*Csf1*	*0.54*	*0.011483*	0.66	0.10244
*Csf2*	1.77	0.337615	1.28	0.73852
*Csf3*	1.93	0.18316	0.95	0.98266
*Ctf1*	0.49	0.131314	1.13	0.73413
*Cx3cl1*	0.57	*0.024194*	0.69	0.14404
*Cxcl1*	2.65	0.164314	1.48	0.2263
*Cxcl10*	1.69	0.232777	1.05	0.94321
*Cxcl11*	1.39	0.368579	0.96	0.96597
*Cxcl12*	*0.44*	*0.009886*	*0.71*	*0.07872*
*Cxcl13*	1.31	0.421716	1.09	0.73564
*Cxcl16*	1.02	0.830846	0.89	0.42328
*Cxcl3*	7.7	0.169696	0.95	0.91528
*Cxcl5*	3.37	0.212199	0.94	0.88692
*Cxcl9*	**5.89**	**0.078554**	**3.3**	**0.06285**
*Fasl*	3.09	0.119071	**2.03**	**0.04219**
*Gpi1*	1.07	0.640631	1.02	0.97433
*Hc*	0.75	0.681503	1.62	0.1135
*Ifna2*	0.93	0.605923	0.44	0.12537
*Ifng*	5.15	0.143744	2.21	0.13276
*Il10*	0.93	0.605923	0.44	0.12537
*Il11*	2.39	0.165436	1	0.82803
*Il12a*	*0.54*	*0.098954*	*0.61*	*0.09123*
*Il12b*	1.47	0.256595	1.62	0.12956
*Il13*	1.09	0.651106	1	0.99484
*Il15*	*0.53*	*0.016916*	*0.73*	*0.07861*
*Il16*	1.01	0.896996	*0.56*	*0.04287*
*Il17a*	1.12	0.484613	*0.35*	*0.08594*
*Il17f*	0.68	0.282624	*0.55*	*0.00207*
*Il18*	**0.46**	**0.070874**	0.87	0.26885
*Il1a*	3.9	0.220118	0.9	0.65997
*Il1b*	3.96	0.172113	1.26	0.38028
*Il1rn*	*0.7*	*0.006867*	*0.73*	*0.03971*
*Il2*	0.94	0.639082	0.48	0.13164
*Il21*	1.6	0.306708	0.44	0.12537
*Il22*	1.87	0.268256	0.54	0.12908
*Il23a*	0.8	0.611696	0.89	0.47794
*Il24*	1.45	0.684595	0.47	0.13168
*Il27*	1.45	0.322021	1.28	0.68824
*Il3*	1.06	0.871143	0.44	0.12537
*Il4*	1.67	0.323998	**2.15**	**0.02924**
*Il5*	0.73	0.547827	0.66	0.31967
*Il6*	3.97	0.227976	1.21	0.40714
*Il7*	0.48	0.180451	0.68	0.13566
*Il9*	0.93	0.605923	0.55	0.16928
*Lif*	1.11	0.576603	*0.51*	*0.01984*
*Lta*	0.65	0.44487	0.55	0.6218
*Ltb*	1.55	0.23314	0.83	0.91681
*Mif*	1.17	0.107953	1.18	0.29987
*Mstn*	1.13	0.736482	0.59	0.21285
*Nodal*	0.93	0.605923	0.52	0.15365
*Osm*	5.53	0.119627	1.56	0.12463
*Pf4*	*0.47*	*0.030614*	*0.63*	*0.05057*
*Ppbp*	1.14	0.986882	1.54	0.55059
*Spp1*	4.12	0.261256	1.77	0.93377
*Tgfb2*	*0.51*	*0.048987*	0.77	0.12043
*Thpo*	1.11	0.432272	1	0.80533
*Tnf*	2.54	0.151012	1.3	0.2214
*Tnfrsf11b*	0.91	0.948453	0.92	0.89644
*Tnfsf10*	1.16	0.779851	1.05	0.97127
*Tnfsf11*	2.2	0.211322	0.96	0.64542
*Tnfsf13b*	0.92	0.651414	0.93	0.44799
*Vegfa*	1.12	0.747986	0.92	0.55534
*Xcl1*	1.1	0.9224	1.28	0.48461

## Supplementary Materials

Supplementary Materials Histological grading criteria used for determination of colitis scores.
