# Supplementary material for: Mast Cells Exert Anti-Inflammatory Effects in an IL10−/− Model of Spontaneous Colitis
Source: Mediators Inflamm. 2018 Apr 17;2018:7817360. doi: 10.1155/2018/7817360 (PMC5932457; doi:10.1155/2018/7817360)
Supplement: Supplementary Materials — Histological grading criteria used for determination of colitis scores. [file 7817360.f1.docx]

| Histopathologic grading criteria | |
| --- | --- |
| Grade |  |
| 0 | Normal tissue |
| 1 | Single focal mononuclear cell infiltrate in lamina propria, minimal epithelial hyperplasia |
| 2 | Multiple focal mononuclear cell infiltrates in lamina propria, mild epithelial hyperplasia |
| 3 | Inflammatory infiltrates involving a large area of mucosa and involving submucosa but not transmural |
| 4 | Transmural inflammation, ulcerations present |
